# Trajectories of Response Inhibition Development in Adolescence

**DOI:** 10.64898/2026.04.03.716386

**Published:** 2026-04-04

**Authors:** Junda Zhu, Colton R. Smith, Clément M. Garin, Xin Maizie Zhou, Finnegan J. Calabro, Beatriz Luna, Christos Constantinidis

**Affiliations:** 1. Program in Neuroscience, Vanderbilt University, Nashville TN 37235 USA; 2. Department of Biomedical Engineering, Vanderbilt University, Nashville TN 37235 USA; 3. Institut des Sciences Cognitives Marc Jeannerod, UMR5229 CNRS Université de Lyon, 69675 Bron Cedex, France; 4. Department of Psychiatry, University of Pittsburgh, Pittsburgh PA 15213 USA; 5. Department of Ophthalmology and Visual Sciences, Vanderbilt University Medical Center, Nashville TN 37232, USA

**Keywords:** monkey, neurophysiology, prefrontal cortex, antisaccade, puberty

## Abstract

Response inhibition is a critical cognitive process that is not fully mature at the time of puberty but continues to improve during adolescence. To understand the neural basis of the maturation process, we obtained longitudinal behavioral, neurophysiological, and imaging data in macaque monkeys as they aged through adolescence. Behavioral performance in several variants of the antisaccade task improved markedly through this period. Neural activity in the prefrontal cortex generally increased, particularly when synchronized to the saccade generation. Trajectories of neural activity and cognitive performance were well predicted by maturation of long-distance white matter tracts connecting the frontal lobe with other brain areas. Our results link the maturation of response inhibition and prefrontal neural activity changes to white matter maturation.

## INTRODUCTION

Improved self-control, including delayed gratification and enhanced response inhibition represent hallmarks of human cognitive development (Mischel et al., 1989; Davidson et al., 2006). By contrast, imperfect impulse control and suboptimal decision making, is a defining characteristic of adolescence (Luna et al., 2004; Casey et al., 2008; Van Leijenhorst et al., 2008; Cauffman et al., 2010). Behavioral tasks that have been devised to test response inhibition, such as the antisaccade task, show marked improvement between adolescence and adulthood (Kramer et al., 2005; Tervo-Clemmens et al., 2023). This period of cognitive enhancement parallels the maturation of the prefrontal cortex (Jernigan et al., 1991; Pfefferbaum et al., 1994; Larsen and Luna, 2018). Anatomical changes in the prefrontal cortex involve changes in gray and white matter volume, surface, and thickness, and myelination of axon fibers within the prefrontal cortex and between the prefrontal cortex and other areas (Luna et al., 2001; Ordaz et al., 2013; Bethlehem et al., 2022). Changes in brain activity that underlie cognitive development through adolescence have been addressed primarily with fMRI studies, which have revealed distinct differences in prefrontal Blood-oxygen level dependent (BOLD) activation between childhood and adulthood in humans performing response inhibition tasks (Padmanabhan et al., 2011; Ordaz et al., 2013).

Understanding the changes that underly improved response inhibition at the level of neural activity has been addressed with animal models and particularly non-human primates that offer a close parallel of response inhibition maturation (Zhou et al., 2016a; Zhou et al., 2016b; Constantinidis and Luna, 2019). Macaque monkeys enter puberty and reach sexual maturity at approximately 3.5 and 5 years, respectively (Plant et al., 2005; Herman et al., 2006). Anatomical studies suggest a protracted period of monkey prefrontal cortical development that continues during the adolescence period (Lewis, 1997; Xu et al., 2020; Kolk and Rakic, 2022). Cortical thickness, surface area, and white matter myelination also appear to follow similar trajectories to those of humans (Kim et al., 2020; Xia et al., 2023; Alldritt and et al., 2024). Biochemical and anatomical changes have also been characterized in the monkey prefrontal cortex from pre-puberty to adulthood, including changes of interneuron morphology and connectivity (Hoftman and Lewis, 2011; Fish et al., 2013).

However, a direct link between cognitive performance in terms of underlying changes in neural activity and concomitant structural brain changes has been lacking. We were therefore motivated to track behavior, neuronal activity, and anatomical imaging measures throughout the adolescence period and relative to key developmental milestones in a cohort of developing monkeys. By determining the relationship of such measurements, we identify the brain mechanisms that can account for the improvement in response inhibition seen during this critical period of life.

## RESULTS

We tracked developmental measures longitudinally in a cohort of four monkeys (Group A, three males, one female) on a quarterly basis, in tandem with neurophysiological recordings and MR imaging, from an age of 3.0±0.1 to 7.1±0.1 years (corresponding to human ages of ~9-21 years). Another four monkeys matched for training time in the task at different time points were used for control comparisons (group B). To align individual growth trajectories on a biological developmental marker rather than chronological age we relied on the closure of the epiphysial growth plate, a well-established indicator of skeletal maturation in humans (Vandewalle et al., 2014; Kovacs et al., 2022). Thus, we defined a “mid-adolescence” age for each monkey as the time of the tibial epiphyseal closure (see methods). The mean mid-adolescence age across individuals was 57.9±3.6 months (corresponding to a human age of ~14.5 years).

### Performance trajectories

**Figure 1 |:**
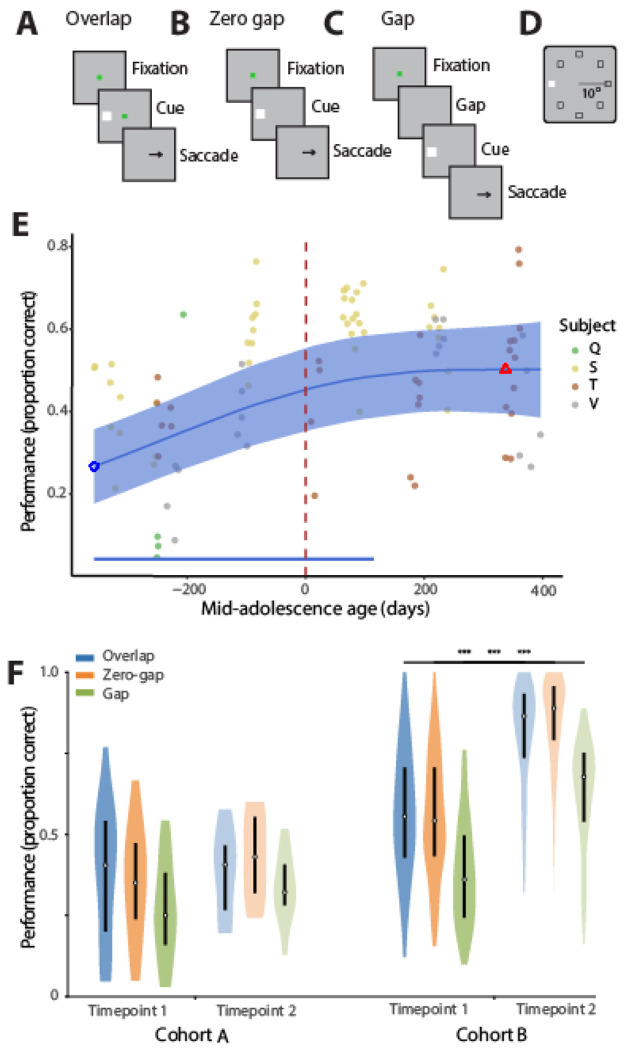
Performance during the antisaccade tasks improves during adolescence. (A-C) Sequence of events in the antisaccade task. The monkey is required to maintain fixation on a fixation point, observe a cue stimulus, and when the fixation point turns off, saccade to a location diametric to the cue. Different task variants were used, in which the fixation point turned off before (A – Overlap variant), simultaneously (B – Zero gap variant), or after the cue appearance (C – Gap variant).(D) Possible locations of the cue stimulus in the screen. (E) Mean performance across all task variants as a function of time, aligned to the mid-adolescence age. Each dot is one session; data from different monkeys are shown in different colors. Blue line shows the GAMM fitted trajectory. Blue circle and red triangle represent minimum and maximum of fitted trajectory. Blue shaded regions denote the 95% confidence intervals (CIs). Dashed vertical line denotes the mid-adolescence age 0. Horizontal bar denotes significant monotonic developmental effect intervals. F. Behavioral performance in the three antisaccade variants in the first two time points for the two cohorts of monkeys (A, tested sequentially through adolescence, and B, for which the second time point of testing was in adulthood).

We evaluated working memory performance with variants of the antisaccade task (Fig. 1), which has been used extensively to evaluate response inhibition in animal (Zhou et al., 2016b) and human studies (Tervo-Clemmens et al., 2023). The monkeys were required to observe a visual cue that could appear at one of eight locations and make an eye movement to a location diametric to the visual stimulus. Behavioral performance was collected at time points spaced ~4 months apart from 3.4 to 6.2 years old. Three variants were used, which differed in their relative timing of the fixation point offset and stimulus onset. Of those, the “overlap” variant (Fig. 1C) was generally the easiest, as it allows subjects to “grasp” their gaze on a visible fixation point at the time of the stimulus appearance, an inappropriate eye movement to which is otherwise more difficult to resist.

Performance in all variants of the task increased as a function of time with highest gains observed early in adolescence (Fig. 1E). The time course of development was similar for different variants, even though the absolute level of performance differed between them (Supplementary Fig. 1).

The monkeys tested later in development had more cumulative exposure to the task as a consequence of our longitudinal experimental design. To evaluate the effect of exposure to the task, we compared the behavioral performance of this group of animals (Group A) with a group of four animals (Group B, all males) that was introduced to the same task at a similar starting age (median 4.3 years) and were trained under the same protocol (Zhou et al., 2016c). After completing their first time point (young stage), their second time point for behavioral testing began 1.6–2.1years later. We compared performance in the three variants of the antisaccade task between the 1^st^ and 2^nd^ testing time points (Fig. 1F). Group A animals exhibited moderate increases in performance between the first two time points that did not reach statistical significance. In contrast, the performance increase in the second time point for cohort B was highly significant for each of the task variants (Mann-Whitney U test, p<10^−10^ in each case). The result indicates that the difference of age during the two testing time points in two groups accounted for a substantial difference in antisaccade performance in the second time points.

### Firing rate changes

**Figure 2 |:**
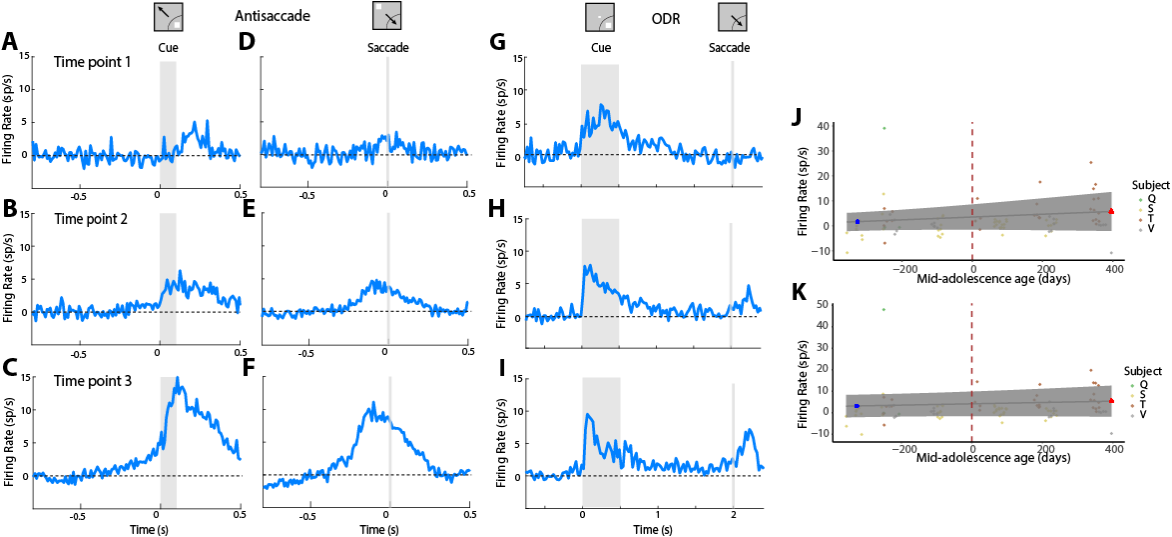
Prefrontal firing rate in the antisaccade tasks increases during adolescence. A-C) Firing rate in the antisaccade task, synchronized on cue onset for three successive developmental stages: time point 1 earliest, n=64 neurons; time point 2, middle, n=122 neurons; time point 3 latest, n=112 neurons). D-F) Same as in A-C, for activity synchronized on the onset of the saccade. G-I) Firing rate in the ODR task from the same neurons, at the same time points. Insets at the top of the figure represent schematically the location of the cue and direction of saccade relative to the preferred location of each neuron used in the population responses, which differed for each neuron. **J)** Firing rate during the cue period, aligned to the mid-adolescence age. Each dot is one session; data from different monkeys are shown in different colors. Blue line shows the GAMM fitted trajectory. Blue circle and red triangle represent minimum and maximum of fitted trajectory. Blue shaded regions denote the 95% confidence intervals (CIs). Dashed vertical line denotes the mid-adolescence age. K) As in J, for the saccadic period.

In agreement with our prior studies (Zhou et al., 2016b; Zhu et al., 2025), we observed that firing rate in the prefrontal cortex generally increased during development. The increase of activity specifically observed in the antisaccade task was evident even after subtracting the baseline fixation rate, and exceeded that observed in the ODR task. Results from data grouped in three time points, for ease of visualization, are shown in Fig. 2. The full distribution of firing rates is shown in Supplementary Fig. 2.

Since measures of firing rate can be skewed by individual differences, we adopted a GAMM approach as well. The results confirmed an increase in firing rate, for both the cue (Fig. 2J) and saccade firing rate (Fig. 2K) during development.

**Figure 4 |:**
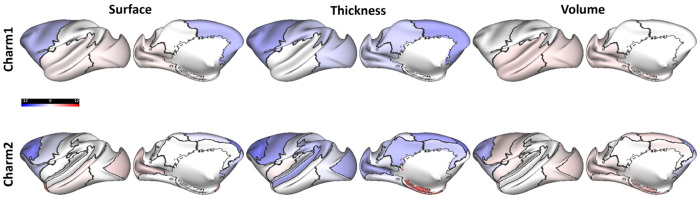
Morphometric developmental changes. Percentage changes in cortical surface area, volume, and thickness during adolescence across brain regions at different levels of segmentation based on the CHARM atlas level 1 (lobar) and level 2 (Jung et al., 2021). Color indicates the percentage change (see color bar) for each region relative to the earliest time points. Changes are projected onto the surface of the NMT template.

This firing rate analysis was based on responses to all neurons. We have previously described however that a critical component of cognitive maturation is vector inversion, the appearance of neural activity representing the location of the saccadic goal, even in neurons that do not otherwise exhibit saccade-driven activity (Zhou et al., 2016b). We wished to examine in more detail the trajectory of maturation of vector inversion. We therefore identified neurons with visual responses alone, in the ODR task. We then examined their responses in the antisaccade task, when the stimulus appeared away from the receptive field. Indeed, we found that activity in this condition progressively increased during development (Fig. 4).

### Correlation with anatomical measures

We examined global cortical gray matter measures to identify which metrics best explain changes in behavioral and neural activity. In a recent study, we reported that frontal lobe cortical thickness in monkeys peaks during adolescence (Zhu et al., 2025). We also observed that the lateral prefrontal cortex (PFC) and motor cortex increase early in development and reach their peak before mid-adolescence, followed by a decline later in adolescence. In the cohort examined here, we observed a decrease in frontal lobe thickness (−4.7%) and surface area (−3.6%) (Fig. 4). This decrease was particularly pronounced in the lateral PFC (surface area: −6.7%, thickness: −7%, and volume: −4.5%). These estimates were based on generalized additive mixed model (GAMM) fits for the three metrics. Most other frontal cortical regions, including the orbitofrontal cortex and anterior cingulate cortex, did not show significant changes in cortical thickness with maturation (Fig. 4). Similarly, most regions in the other lobes (parietal, temporal, occipital) did not show significant variations (below 3%) across the three metrics, except for the medial temporal area, which showed an increase in volume by 4% and thickness by 8.8%

**Figure 5 |:**
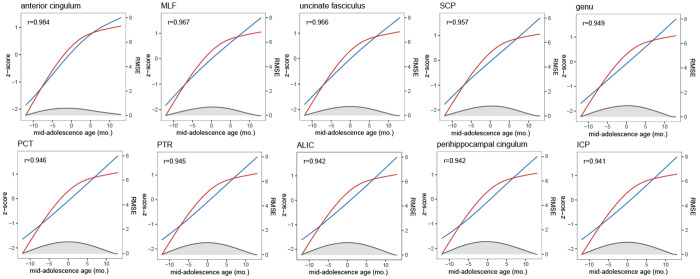
Correlation between performance and FA. Plots of 10 white matter tracks with highest correlation between performance (red curve) and Fractional Anisotropy (FA – blue curve). Shaded area represents root mean squared error.

On the other hand, behavioral trajectories for three different trial-type subsets closely matched the maturation of white matter tracts. Since the performance across all task types was highly correlated with individual task variants, we pooled results from all tasks together. The overall performance trajectory is highly representative of the trial-type specific curves (Pearson correlation values of the overall trajectory and individual task trajectories was r > 0.99 in all cases). We examined in detail the white matter tract maturation that most closely paralleled the trajectory of working memory performance improvement and prefrontal cortical activity changes (Fig. 5). We thus performed correlation analyses between the tracts we identified and the behavioral performance of the antisaccade task. We analyzed Fractional Anisotropy (FA) for 53 identified tracts. The performance in the antisaccade task showed strong trajectory alignment with the FA of most tracts (median |r|=0.9412, median RMSE =0.7083). Examples of high-alignment tracts included the Anterior Cingulum (r=0.9836, RMSE=0.4228) and Medial Longitudinal Fasciculus (MLF; r=0.9673, RMSE=0.4722). In parallel, Radial Diffusivity (RD) showed predominantly inverse alignment with behavior (median r =−0.9387; median RMSE = 0.6771).

## DISCUSSION

Our longitudinal study provides direct evidence for coordinated development of response inhibition, prefrontal neural activity, and white matter maturation during primate adolescence. Monkeys, as humans, exhibited substantial variability in development, we therefore relied on objective biomarkers of physical development that allowed us to align growth trajectories of different individuals. We then analyzed multimodal data that allowed us to tie improvements in behavioral to neural activity and structural brain changes. Behavioral performance in several variants of the antisaccade task improved substantially through the adolescent period, accompanied by progressive increases in prefrontal cortical firing rates, particularly around the time of saccade generation. Most importantly, these behavioral and neural trajectories were tightly aligned with the maturation of long-distance white matter tracts linking the frontal lobe with distributed cortical areas. These results identify white matter maturation as a key structural substrate underlying the enhancement of the ability to resist the prepotent influence of an external stimulus during this critical developmental period.

### Maturation of response inhibition

Human cognitive ability improves significantly during adolescence, particularly in domains requiring response inhibition and self-control (Simmonds et al., 2017; Montez et al., 2019). The antisaccade task shows marked developmental improvements from childhood through adolescence in humans (Kramer et al., 2005; Davidson et al., 2006). Deficits in antisaccade performance characterize childhood neurodevelopmental conditions such as ADHD(Munoz et al., 2003) and are prominent in mental illnesses that manifest in early adulthood, such as schizophrenia (McDowell et al., 2002; Smyrnis et al., 2004). Understanding the neural mechanisms underlying the normative development of response inhibition is therefore of considerable basic science as well as clinical importance.

Our analysis of behavioral performance revealed that adult monkeys improve not only in accuracy but also in reaction time, effectively defeating the typical speed-accuracy tradeoff (Zhu et al., 2024). This pattern, which mirrors findings in humans (Salinas et al., 2019), reflects a mature capacity to suppress rapid, stimulus-driven responses and execute more deliberate, goal-directed eye movements. The observation that saccades generated early after stimulus onset are much more likely to result in errors, as the rapid stimulus transient captures gaze, is consistent with the hypothesis that developmental improvements reflect enhanced inhibitory control over reflexive responses. The progressive increase in ability to resist stimulus-driven capture and generate appropriate delayed responses constitutes a major component of cognitive maturation during adolescence.

### Neural activity substrates of cognitive maturation

In accordance with our prior studies (Zhou et al., 2016c; Zhu et al., 2025) we observed a progressive increase in prefrontal cortical firing rates during development in the antisaccade task that exceeded the changes observed in the control oculomotor delayed response task. The result suggests that the changes are specifically related to the cognitive demands of response inhibition rather than reflecting general developmental changes in motor processing.

A critical component of this neural maturation is the emergence of vector inversion, the representation of the saccadic goal in neurons that do not otherwise exhibit saccade-driven activity (Zhou et al., 2016a; Zhou et al., 2016b). Our analysis revealed that activity in neurons with purely visual responses (identified based on the pattern of responses in the ODR task following visual stimuli out of these neurons’ receptive fields ) progressively increased during development. This pattern indicates a refinement of neural computations underlying response inhibition, where neurons increasingly encode task-relevant information beyond their baseline sensory selectivity. Fixation period activity generally increased during the period of development (Zhu et al., 2025). While often dismissed as “baseline” or background activity, substantial evidence suggests that neurons active during fixation play a functional role in representing task parameters and suppressing eye movements (Zhou and Constantinidis, 2017; Riley et al., 2018).

How neural activity changes produce behavioral outputs is often opaque, however artificial neural networks have been used successfully to uncover the underlying computations (Masse et al., 2019; Xie et al., 2022). Increases in activity in the antisaccade task have been thus recapitulated in artificial recurrent neural networks, which show increases in unit activity as the network performance on antisaccade tasks improves (Liu et al., 2021). This computational finding suggests that the observed activity changes in neural maturation reflects fundamental principles of optimization in neural systems.

### Structural Brain Development

Recent structural imaging studies have documented trajectories of age-related brain changes across large human cohorts (Bethlehem et al., 2022) and monkey populations (Alldritt and et al., 2024). A hallmark of brain development is the decrease in cortical volume and thickness after childhood (Gogtay et al., 2004). This process is thought to be driven by pruning of infrequently used synapses (Bourgeois et al., 1994), though apparent decreases in thickness may also reflect myelination of gray matter (Natu et al., 2019). Synaptic pruning has been shown to enhance neural computations, leading to enhanced stability and improved efficiency of neural representations (Averbeck, 2022; Liuzzi et al., 2023).

In our cohort, we observed decreases in frontal lobe cortical thickness (−4.7%) and surface area (−3.6%), with pronounced changes in the lateral prefrontal cortex (surface area: −6.7%, thickness: −7%, volume: −4.5%). Our findings revealed an even more striking association between white matter maturation and improvements in both behavioral performance and prefrontal neural activity. We examined fractional anisotropy (FA) in 55 identified white matter tracts and found that they showed strong trajectory alignment with antisaccade performance. Example high-alignment tracts included the anterior cingulum and medial longitudinal fasciculus, tracts that interconnect key cortical and subcortical regions involved in eye movement control and executive function. Radial diffusivity (RD) showed predominantly inverse alignment with performance, consistent with the interpretation that increasing white matter integrity underlies cognitive maturation.

These findings align with extensive literature highlighting the protracted maturation of white matter pathways in humans, which continues well into early adulthood (Lebel et al., 2012; Simmonds et al., 2014). Our results suggest that the adolescent period is characterized by a critical phase of white matter maturation that directly enables the integration of distributed cortical networks required for sophisticated cognitive control. The high correlation between white matter measures and both behavioral performance and neural activity trajectories suggests that enhanced white matter integrity facilitates more efficient communication among prefrontal regions and between prefrontal and subcortical structures, thereby improving the neural computations underlying response inhibition.

### Limitations and Future Directions

Several limitations merit acknowledgment in interpreting our findings. Although non-human primates provide the closest animal models to humans, there remain important differences between species that may limit generalization of our conclusions. Specifically, while monkeys undergo developmental trajectories broadly similar to humans, the rate and timing of cognitive development differ between species. Additionally, our neurophysiological recordings were restricted to the prefrontal cortex, motivated by its well-documented role in cognitive control and the specializations of neurons within this region (Zhou et al., 2012; Zhou et al., 2016a; Jaffe and Constantinidis, 2021). However, response inhibition is fundamentally a network-level phenomenon involving distributed circuitry spanning prefrontal, parietal, subcortical, and brainstem regions. The superior colliculus and posterior parietal cortex play a critical role in oculomotor control and inhibition; future work examining how development modulates activity in these distributed networks would provide a more complete mechanistic understanding (Johnston et al., 2007; Qi et al., 2010). Finally, our analysis of neural activity here relied only on single-neuron responses; though changes with increased performance have been described in coordinated neural activation reflected in local field potentials (Wang et al., 2022) and synchronous spiking revealed with methods such as spike-count correlation (Qi and Constantinidis, 2012). Follow up studies can provide a more global picture of factors contributing to response inhibition maturation.

## METHODS

### Subjects

Behavioral, imaging, and neurophysiological recordings were obtained from a total of eight (7 male, 1 female) rhesus monkeys (*Macaca mulatta*). Most data presented here belonged to Cohort A which included four monkeys (3 males, 1 female) and was tracked throughout adolescence. Cohort B included another four monkeys (4 males) and was tested at two time points (early adolescence, adulthood), providing a control for training exposure. All surgical and animal use procedures were reviewed and approved by the Institutional Animal Care and Use Committees of Wake Forest University and Vanderbilt University, in accordance with the U.S. Public Health Service Policy on Humane Care and Use of Laboratory Animals and the National Research Council’s Guide for the Care and Use of Laboratory Animals.

### Developmental markers

We tracked developmental measures of Cohort A monkeys on a quarterly basis before, during, and after neurophysiological recordings. Monkeys of this cohort were first obtained at an age of 2.3 – 2.9 years from a commercial breeding company (Alpha Genesis, Yemassee, South Carolina) where they were mother-reared and lived in species-typical social groups. Once in the laboratory, they were housed in groups of two-three animals, in view of each other, and other conspecifics. To determine each monkey’s developmental progress, we relied primarily on skeletal assessment to best capture physical development (Cheng et al., 2021; Kovacs et al., 2022) and align the growth trajectories of different individuals. Using these measures, we defined a mid-adolescence age for each monkey, defined as the time of each monkey’s distal tibial epiphyseal closure, as observed by veterinary professionals evaluating the X-rays, blind to findings of other aspects of the study.

### Behavioral Tasks

All monkeys were trained to perform the Oculomotor Delayed Response (ODR) Task and antisaccade tasks. The ODR task is a spatial working memory task requiring subjects to remember the location of a cue stimulus flashed on a screen for 0.5 s. The cue was a 1° white square stimulus that could appear at one of eight locations arranged on a circle of 10° eccentricity. After a 1.5 s delay period, the fixation point was extinguished and the monkey was trained to make an eye movement to the remembered location of the cue within 0.6 s. In the antisaccade task, each trial starts with the monkey fixating a central green point on the screen. After 1s fixation, the cue appears, consisting again of a 1° white square stimulus at one of the same eight locations arranged on a circle of 10° eccentricity for 0.1 s. The monkey is required to make a saccade at the location diametric to the cue. The saccade needed to terminate on a 5–6° radius window centered on the stimulus (within 3–4° from the edge of the stimulus), and the monkey was required to hold fixation within this window for 0.1 s. Animals were rewarded with fruit juice for successful completion of a trial. Eye position was monitored with an infrared eye tracking system (ISCAN, RK-716; ISCAN, Burlington, MA).

We used three different variants for the antisaccade task: overlap, zero gap, and gap, differing in the sequence of the cue onset relative to the fixation point offset (Fig. 1). In the overlap condition, the cue appears first, and then and fixation point and cue are simultaneously extinguished. In the zero gap condition, the fixation offset and the cue onset occur at the same time. In the gap condition, the fixation turns off and a 100 ms blank screen is inserted before the cue onset. The monkeys were trained in the antisaccade task with the stimulus appearing at any of the eight stimulus locations also used in the ODR task, however during recordings, four possible cue locations for each condition were used, involving either the cardinal axes or the diagonal axes, so there were 3 x 4 types of trials in each block of trials. The sequence of these 12 trials was randomized in each block. During each recording session, monkeys first performed the ODR task which helped determine the receptive field of neurons recorded online, and then performed the antisaccade task.

The visual stimulus display, monitoring of eye position, and synchronization of stimuli with neurophysiological data were performed using in-house software, and implemented with MATLAB (Meyer and Constantinidis, 2005). We analyzed performance in each variant of the antisaccade task by expressing performance as the overall percentage of trials that resulted in correct responses.

### Surgery and Neurophysiology

The monkeys were initially trained in the tasks mentioned above before their neurophysiological recordings. They were naïve to behavioral training or task execution of any kind prior to the behavioral training. After the animals of cohort A had reached asymptotic performance in the behavioral tasks for the first time, we implanted a 20-mm diameter recording cylinder over the prefrontal cortex of each monkey. Localization of the recording cylinder was based on MR imaging, processed with the BrainSight system (Rogue Research, Montreal, Canada). Recordings in each time point were collected with glass or epoxylite coated Tungsten electrodes with a diameter of 250 μm and an impedance of 4 MΩ at 1 KHz (FHC Bowdoin, ME). Electrode penetrations within the cylinder were placed with a stereotaxic grid system (Crist Instruments, Inc, Hagerstown, MD) to ensure we precisely and evenly sample from the region of interest at each time point, without visiting the exact same grid location, to avoid accumulated damage. Within this grid, neurons were sampled in an unbiased fashion, recording from all neurons encountered in our penetrations, without an effort to select some neurons based on any functional properties. Electrical signals recorded from the brain were amplified, band-pass filtered between 500 Hz and 8 kHz, and stored through a modular data acquisition system at 25 μs resolution (APM system, FHC, Bowdoin, ME).

Recordings were obtained and analyzed from areas 8a and 46 of the dorsolateral prefrontal cortex. Neurons were not pre-screened prior to collection; we recorded from all neurons isolated from our electrodes. Recorded spike waveforms were sorted into separate units using a semi-automated cluster analysis method based on the KlustaKwik algorithm (Harris et al., 2000). Neurons for which at least 4 correct trials in every stimulus condition were available in the ODR task were used in the following analyses.

Neurophysiological recordings were obtained from the monkeys of cohort A at time points spaced approximately 3 months apart from 3.4 to 6.2 years old. Each behavioral time point of each animal contained an average of 19 sessions. Between time points, the animals were returned to their colony and were not tested or trained in any task until their next time point.

### MRI acquisition and preprocessing

Structural MRIs were collected from the monkeys of cohort A every 3 months from 2.8 years (34 months) of age to 5.8 years (69 months) of age. In preparation for the MRI scan, anesthesia was induced using ketamine (5–10mg/kg) and dexmedetomidine (0.015mg/kg), and was maintained using isoflurane. The animals were intubated and artificially ventilated at about 20 breaths per minute. Expired CO_2_ was monitored and maintained between 35 and 45 mmHg. Animals were scanned under isoflurane anesthesia at 1%–1.5%. Heart rate and oxygen saturation levels were monitored using a pulse oximeter. Their body temperature was maintained using warm blankets. The MRI system was a 3 Tesla Siemens MAGNETOM Skyra (Siemens Healthcare, Erlangen, Germany). Anatomical images were acquired using a T1-weighted MPRAGE sequence: TR = 2700 ms, TE = 3.32 ms, inversion time = 880, FOV = 128 × 128 mm, 192 slices of 0.5 mm thickness, resolution = 0.5 mm isotropic. Resting state time series data were also acquired using a multiband EPI sequence: TR = 700 ms, TE = 32.0 ms, flip angle = 52°, repetitions = 700, FOV = 128 × 128 mm, 32 slices, resolution = 2 mm isotropic.

Spatial pre-processing was performed using the EvoDevo NeuroImaging Explorer (EDNiX) pipeline (https://github.com/garincle/EDNiX) as described before (Zhu et al., 2025). This relied on functions from AFNI, ANTs (Avants et al., 2009), FSL (Jenkinson et al., 2012), FreeSurfer (Fischl, 2012) and Connectome Workbench (Marcus et al., 2013) for inhomogeneity correction, spatial and surface registration to a standardized space. A high-resolution NMT template (NIH Macaque Template) as well as the CHARM (Jung et al., 2021) and SARM (Hartig et al., 2021) atlas segmentations were registered to the study template. Individual anatomical T1 images (T1^n^) were registered to their T1^last^ and each T1^last^ was registered to the study template. The two movement parameters (T1^n^ to T1^last^ and T1^last^ to study template) were combined to register T1^n^ to the study template. Inversion of these movement parameters was used to register the NMT atlases to the individual T1^n^ images. The volumes (in mm^3^) were calculated using the AFI function “3dhistog”. Surface and thickness were reconstructed using the Connectome Workbench. Figures were produced using the Connectome Workbench and nilearn (Abraham et al., 2014). Surface, thickness, and volume measurements of cortical and subcortical structures were performed on the hemisphere opposite to the one where recordings were performed.

### DTI preprocessing

Diffusion Tensor Imaging (DTI) data were acquired in pairs with a reversed phase encoding direction in the second scan (e.g., PA vs AP). A diffusion-weighted spin-echo echo-planar imaging sequence was utilized to obtain 82 whole-brain slices of 2mm thickness in 30 directions. Data were processed for analysis using MRtrix3 (Tournier et al., 2019) and the Oxford Centre of fMRI of the Brain Software Library (FSL). The raw DICOM images acquired from the scanner were converted to NIFTI format using dcm2nii, and the corresponding bval and bvec files containing information pertinent to the diffusion gradient were combined across scans. Images were then denoised (“dwidenoise”) and mean b=0 images were calculated. Following this, susceptibility induced and eddy current distortion was corrected using FSL (“TOPUP”) and (“eddycorrect”) respectively. A tensor model was fitted to each voxel (“dwi2tensor”) and fractional anisotropy (FA) maps were calculated (“tensor2metric”). A mask was also created from the T1-weighted image (“bet”), segmenting the brain and non-brain tissue from the whole head.

Each FA image was then registered to the subject’s respective skull-stripped T1-weighted image by affine transformation. These co-registered images were subsequently registered to a diffusion-tensor-based white matter atlas for rhesus macaques (Zakszewski et al., 2014). This allowed for a group analysis of several parameters: 1) the directionality of water diffusion within white matter tissue (Fractional Anisotropy - FA), 2) the mean apparent diffusion coefficient of the diffusion tensor (mean diffusivity – MD), 3) the principal eigenvalue, or diffusion parallel to the principal axis of diffusion (axial diffusivity - AD), and 4) the mean of the two non-principal eigenvalues, or the diffusion perpendicular to the principal axis of diffusion (radial diffusivity - RD). The MD, AD, and RD maps were derived from the diffusion tensor using the same method employed for generating the FA maps. After initial processing, a whole-brain region-of-interest (ROI) analysis was performed, with 53 white matter tracts from the DTI atlas selected. Left and right hemispheres were separately calculated and averaged together.

### Generalized Additive Mixed Models

To characterize adolescent development of behavior, activity, and brain structural measures in our longitudinal sample, we used generalized additive mixed models (Wood, 2012). All GAMM analyses were implemented using the mgcv package for R, each with a smooth function of mid-adolescence age as a covariate, using a thin plate regression spline basis to estimate this smooth function. Random effects in each GAMM included subject-specific intercepts and slopes for mid-adolescence age. For each measurement, we fit a generalized additive mixed model (GAMM) for each outcome variable using mgcv::gamm with a smooth term for maturation: s(mid_adolescence age, k=5, bs=‘cs’, fx=FALSE, REML estimation, and subject-level random effects (~1 + mid_adolescence age by subject). Models were fit using complete cases per outcome and predictor. Predicted trajectories were generated on a fixed maturation grid with 100 grid points. Trajectory similarity was quantified by z-scoring each predicted curve and computing Pearson correlation (r) across the shared grid. Shape mismatch was quantified as RMSE between baseline-shifted absolute trajectories. For those models for which there was a statistically significant fixed effect, the gratia package for R (Simpson and Singmann, 2018) was used to conduct exploratory post-hoc analyses to identify significant periods of developmental change. Specifically, the derivatives of each estimated smooth function of age were approximated using the method of finite differences, and a simultaneous 95% confidence. Because we tested the effect of age on several outcome measures, in separate GAMMs, we applied a false discovery rate (FDR) correction (Benjamini–Hochberg) to control for multiple comparisons. The reported p-values reflect the adjusted significance levels unless otherwise specified.

### Correlation between trajectories

To evaluate similarity between developmental trajectories, we calculated the correlation between the GAMM predictions of different measures. To ensure consistency and comparability, the predictor values (mid-adolescence age) were evenly sampled at 100 intervals between the earliest and latest time points for behavioral data. Each curve was then normalized using z-score normalization.

We calculated two key metrics: the Pearson correlation coefficient (r) and the Root Mean Square Error (RMSE). Before calculating RMSE, each normalized curve was shifted such that the starting point was aligned to zero. This was done by subtracting the starting value of the curve. After shifting, the absolute values of the data points were taken to ensure uniformity in the direction of both curves, facilitating a comparable visualization using RMSE between positively and negatively correlated pair of curves. The RMSE was calculated as:

$$RMSE-\sqrt{\frac{1}{n}\sum_{i=1}^{n}{\left(\left|X_{i}\right|-\left|Y_{i}\right|\right)}^{2}}$$

where $\left|X_{i}\right|$ and $\left|Y_{i}\right|$ are the absolute values of the shifted and normalized data points from the respective curves.

We conducted a permutation test with a maxT approach to assess the statistical significance of the observed correlation coefficients. We randomly permuted *X* and *Y* to recalculate the correlation coefficient across 1,000 permutations to simulate the null hypothesis of no correlation. The maxT p-value was calculated to determine the probability of observing a correlation as extreme as the detected one, or more extreme, under the null hypothesis.

## Supplementary Material

1

## Figures and Tables

**Figure 3 |:**
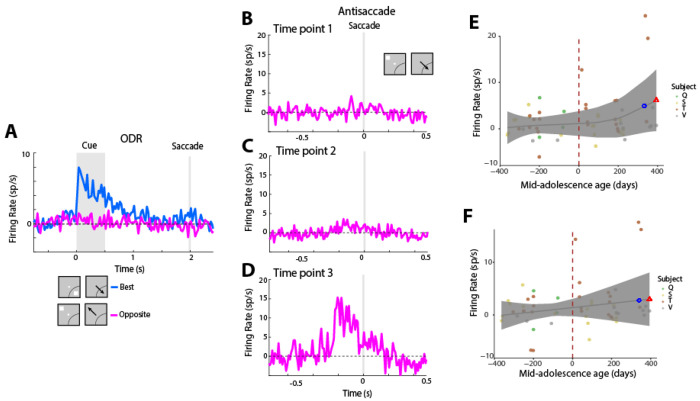
Firing rate of visual neurons. **A)** Firing rate in the ODR task for neurons with only visual activity. Insets at the bottom of the figure represent schematically the location of the cue and direction of saccade relative to the preferred location of each neuron used in the population responses, which differed for each neuron (n=72 neurons). B-D). Firing rate in the antisaccade task synchronized on saccade delivery, when the cue appeared out of the receptive field for three successive time points. Conventions are the same as in Fig. 2. E) GAMM for firing rate aligned to the cue as a function of age relative to mid-adolescence age. F) As in E, for firing rate aligned to the saccade.

## Data Availability

Data for the current study will be made available through Zenodo upon acceptance of the paper

## CODE AVAILABILITY

The code used to process the results and generate the figures will be made available at Github upon acceptance of the paper
