# Supplementary material for: Trajectories of Response Inhibition Development in Adolescence
Source: bioRxiv. 2026 Jun 5:2026.04.03.716386. Originally published 2026 Apr 4. Preprint. [Version 2] doi: 10.64898/2026.04.03.716386 (PMC13060240; doi:10.64898/2026.04.03.716386)
Supplement: Supplement 1 [file NIHPP2026.04.03.716386v2-supplement-1.pdf]

bioRxiv preprint doi: https://doi.org/10.64898/2026.04.03.716386; this version posted June 5, 2026. The copyright holder for this preprint (which was not certified by peer review) is the author/funder, who has granted bioRxiv a license to display the preprint in perpetuity. It is made available under a CC-BY 4.0 International license.

SUPPLEMENTARY INFORMATION

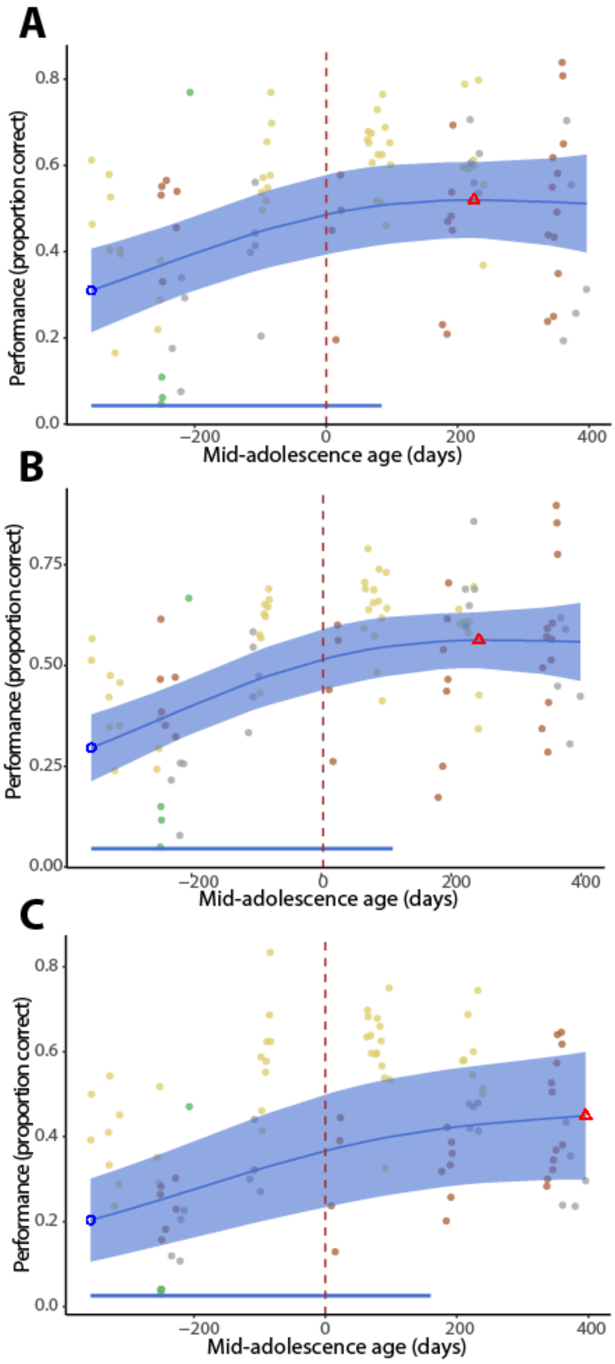

Supplementary Figure 1. Performance in the three different variants of the antisaccade task.. (A) Overlap variant. (B) Zero gap-variant. (C) Gap-variant. Conventions are the same as in Fig. 1E. Different colors represent the same subjects, as in Fig. 1E.

bioRxiv preprint doi: https://doi.org/10.64898/2026.04.03.716386; this version posted June 5, 2026. The copyright holder for this preprint (which was not certified by peer review) is the author/funder, who has granted bioRxiv a license to display the preprint in perpetuity. It is made available under a CC-BY 4.0 International license.

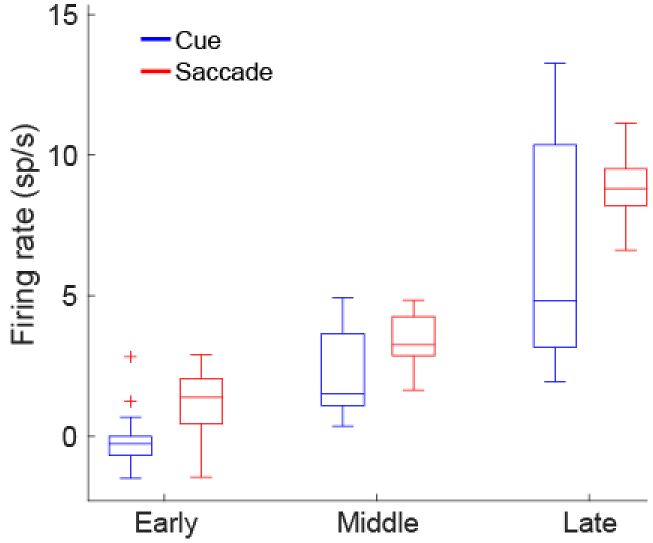

Supplementary Figure 2. Distribution of firing rates aligned to the cue presentation and saccade, after subtracting the baseline firing rate at each of the early, middle, and late subsamples.

bioRxiv preprint doi: https://doi.org/10.64898/2026.04.03.716386; this version posted June 5, 2026. The copyright holder for this preprint (which was not certified by peer review) is the author/funder, who has granted bioRxiv a license to display the preprint in perpetuity. It is made available under a CC-BY 4.0 International license.

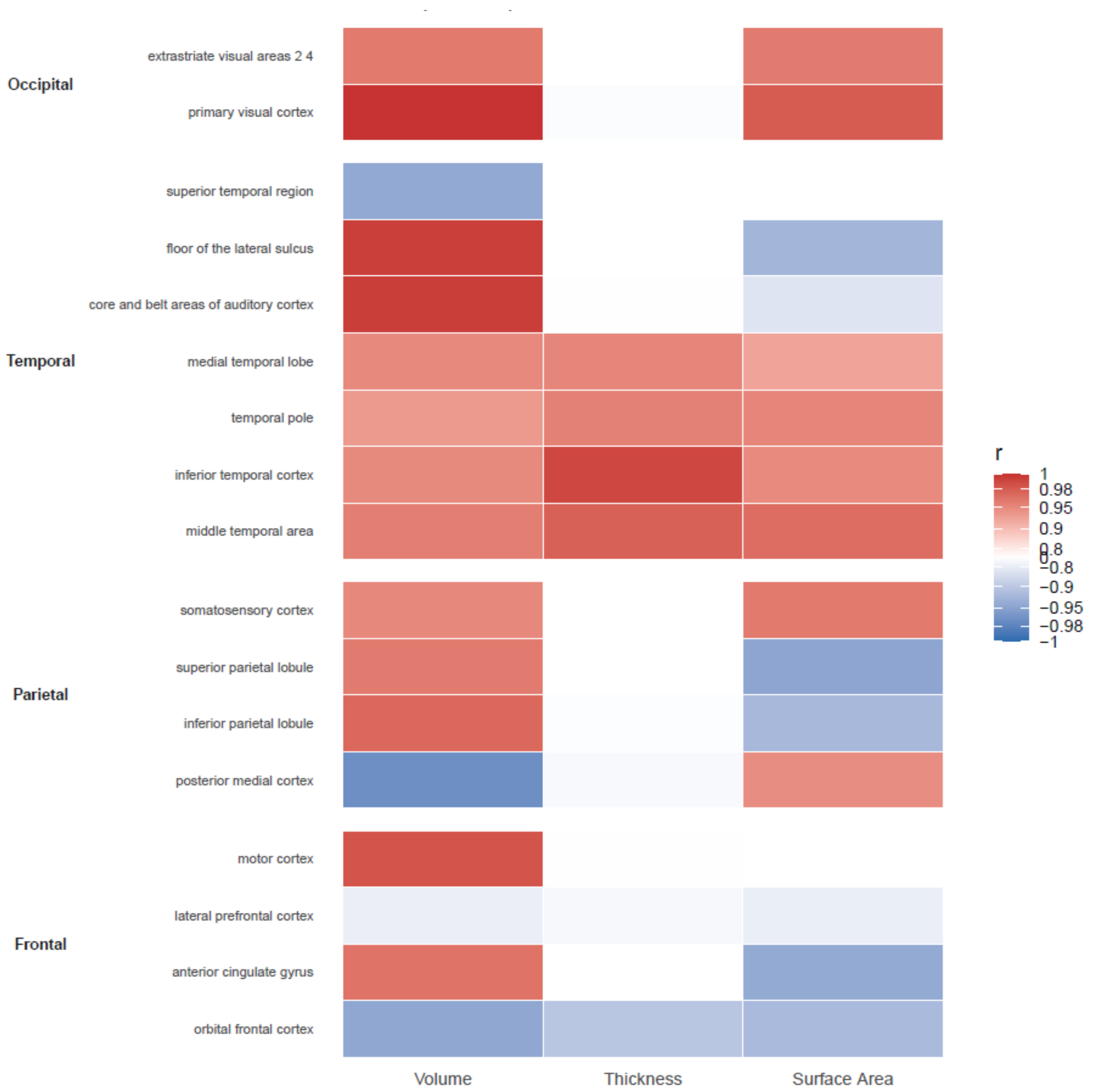

Supplementary Figure 3. Correlation between behavior, averaged across all task variants, and structural brain measures of volume, thickness, and surface area.

bioRxiv preprint doi: https://doi.org/10.64898/2026.04.03.716386; this version posted June 5, 2026. The copyright holder for this preprint (which was not certified by peer review) is the author/funder, who has granted bioRxiv a license to display the preprint in perpetuity. It is made available under a CC-BY 4.0 International license.

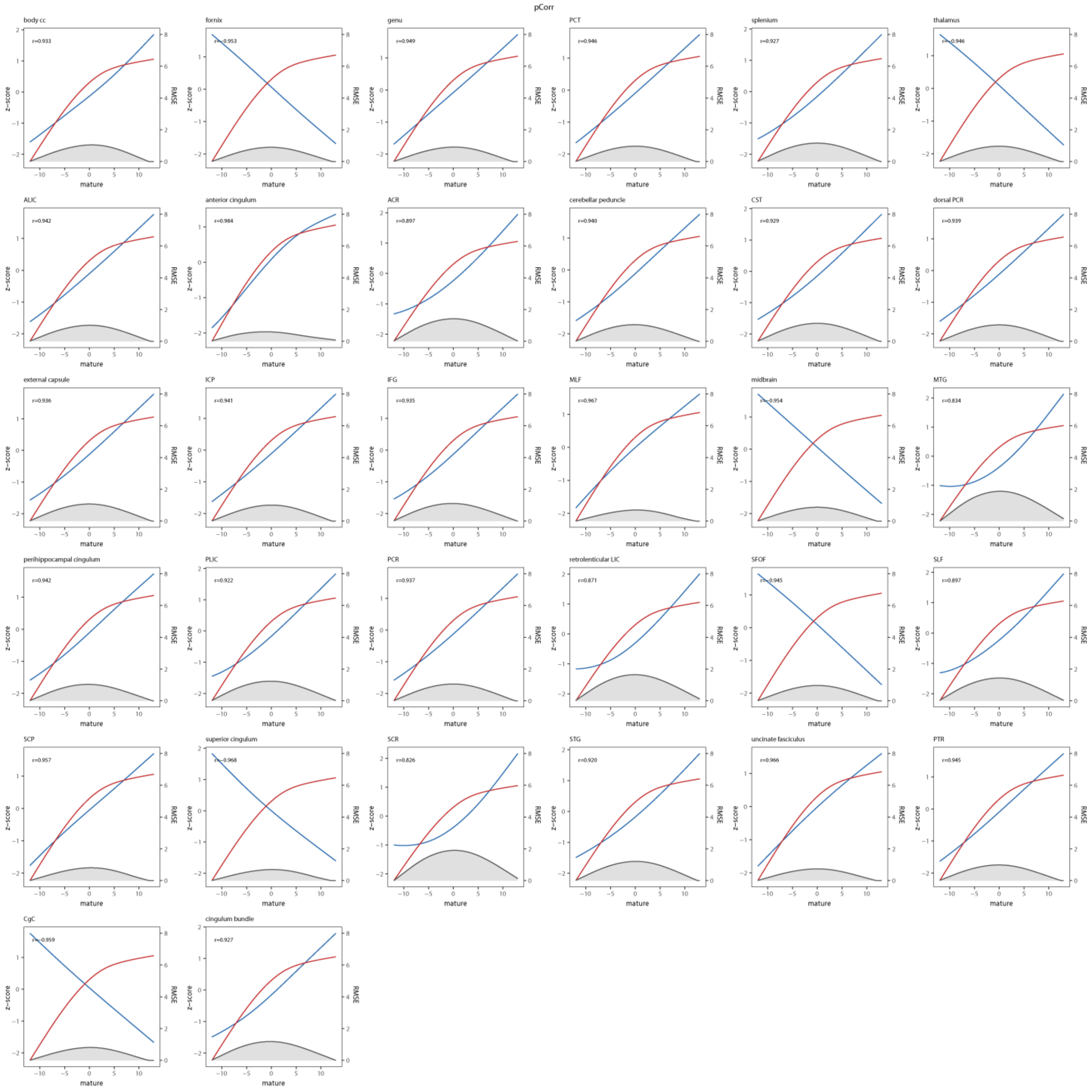

Supplementary Figure 4. Correlation between performance and FA. Plots of all available white matter tracks showing correlation between performance (red curve) and Fractional Anisotropy (FA – blue curve). Shaded area represents root mean squared error.

bioRxiv preprint doi: https://doi.org/10.64898/2026.04.03.716386; this version posted June 5, 2026. The copyright holder for this preprint (which was not certified by peer review) is the author/funder, who has granted bioRxiv a license to display the preprint in perpetuity. It is made available under a CC-BY 4.0 International license.

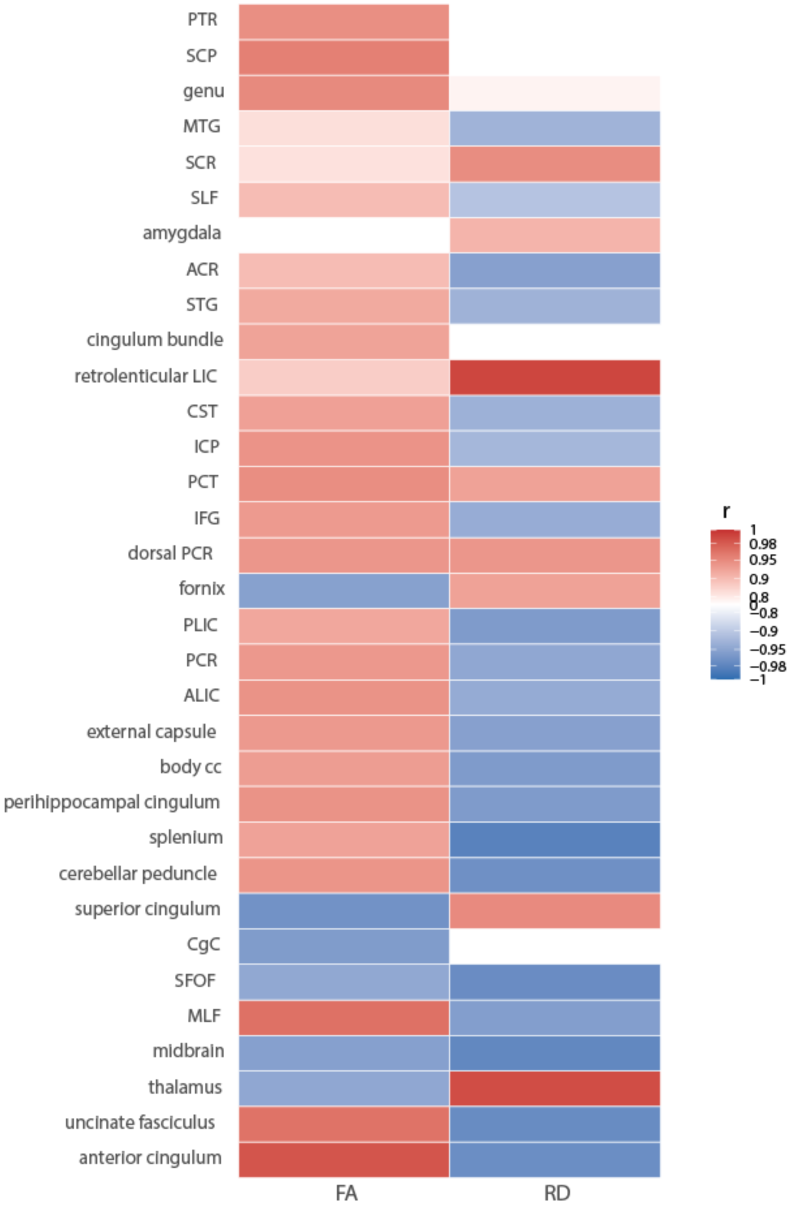

Supplementary Figure 5. Correlation between behavior, averaged across all task variants, and FA/RD trajectory.
