# Supplementary material for: Trajectories of Response Inhibition Development in Adolescence
Source: bioRxiv. 2026 Apr 4:2026.04.03.716386. Preprint. [Version 1] doi: 10.64898/2026.04.03.716386 (PMC13060240; doi:10.64898/2026.04.03.716386)
Supplement: Supplementary file 1 [file NIHPP2026.04.03.716386v1-supplement-1.pdf]

bioRxiv preprint doi: https://doi.org/10.64898/2026.04.03.716386; this version posted April 5, 2026. The copyright holder for this preprint (which was not certified by peer review) is the author/funder, who has granted bioRxiv a license to display the preprint in perpetuity. It is made available under aCC-BY 4.0 International license.

SUPPLEMENTARY INFORMATION

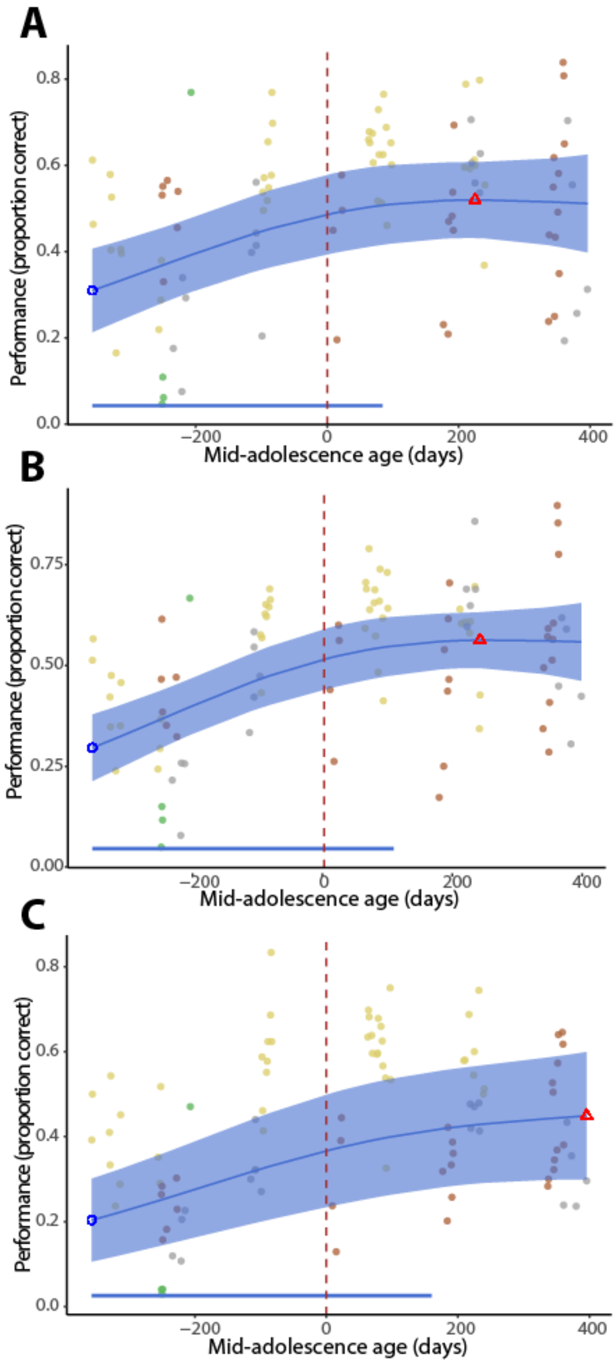

Supplementary Figure 2. Performance in the three different variants of the antisaccade task.. (A) Overlap variant. (B) Zero gap-variant. (C) Gap-variant. Conventions are the same as in Fig. 1E. Different colors represent the same subjects, as in Fig. 1E.

bioRxiv preprint doi: https://doi.org/10.64898/2026.04.03.716386; this version posted April 5, 2026. The copyright holder for this preprint (which was not certified by peer review) is the author/funder, who has granted bioRxiv a license to display the preprint in perpetuity. It is made available under aCC-BY 4.0 International license.

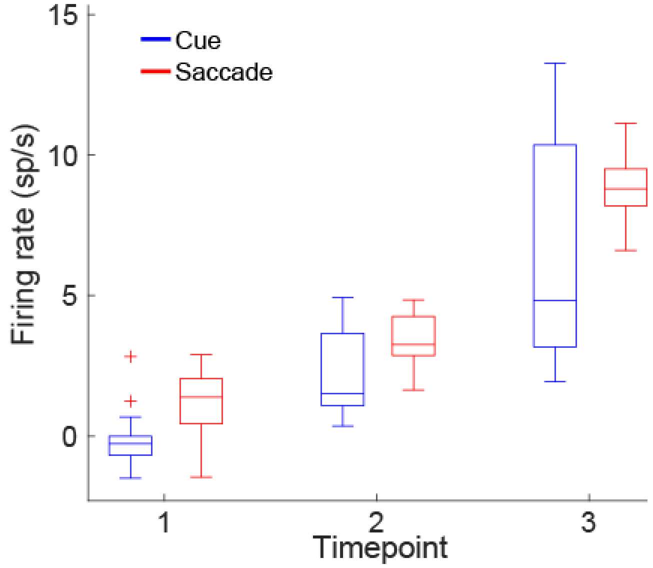

Supplementary Figure 2. Distribution of firing rates aligned to the cue presentation and saccade, after subtracting the baseline firing rate at each of the three time points.

bioRxiv preprint doi: https://doi.org/10.64898/2026.04.03.716386; this version posted April 5, 2026. The copyright holder for this preprint (which was not certified by peer review) is the author/funder, who has granted bioRxiv a license to display the preprint in perpetuity. It is made available under aCC-BY 4.0 International license.

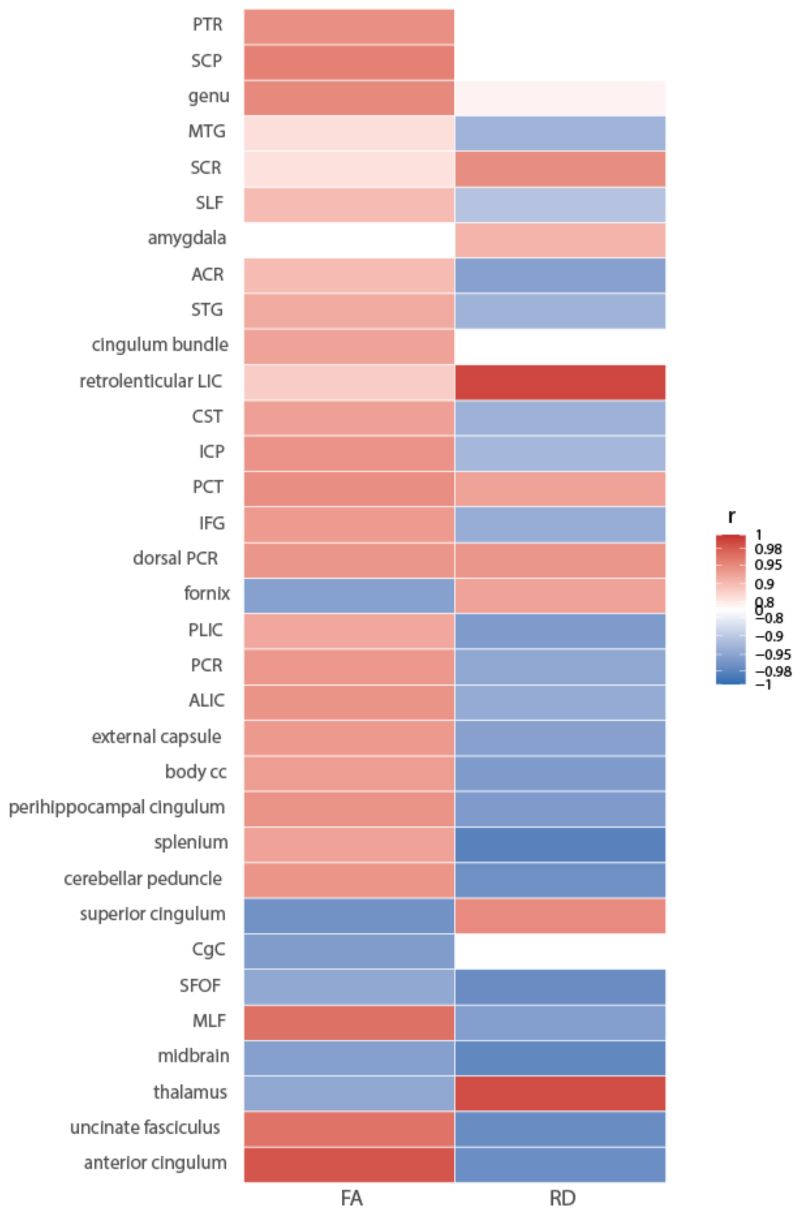

Supplementary Figure 3. Correlation between behavior, averaged across all task variants, and FA/RD trajectory.
